# Inhibition of Parathyroid Hormone Secretion by Caffeine in Human Parathyroid Cells

**DOI:** 10.1210/jc.2013-1466

**Published:** 2013-06-20

**Authors:** Ming Lu, Lars-Ove Farnebo, Robert Bränström, Catharina Larsson

**Affiliations:** Departments of Molecular Medicine and Surgery (L.-O.F., R.B.), and Oncology-Pathology (M.L., C.L.), Karolinska Institutet, SE-171 76 Stockholm, Sweden; and Department of Geriatric Endocrinology (M.L.), First Affiliated Hospital of Guangxi Medical University, Nanning 520021, China

## Abstract

**Context and Objective:**

Caffeine is a highly consumed psychoactive substance present in our daily drinks. Independent studies have reported associations between caffeine consumption, low bone mineral density, and urinary calcium loss, as well as impaired bone development in vitro and in vivo. Calcium (Ca^2+^), vitamin D, and PTH are critical regulators of bone remodeling. A possible association between caffeine and parathyroid gland function has been suggested in the literature.

**Design, Setting, and Patients:**

Effects of caffeine on PTH secretion and Ca^2+^ levels were determined by batch incubation and Fura-2, respectively, in pathological parathyroid cells. Protein expressions were studied by Western blot and immunohistochemistry in normal and parathyroid adenoma tissues. Alterations in gene expressions of adenosine receptor A1 (*ADORA1*) and A2 (*ADORA2A*) and *PTH* were quantified by PCR; intracellular cAMP levels and protein kinase A activity were analyzed by an antibody-based assay.

**Results:**

We studied physiological concentrations of caffeine ranging from 1 to 50 μm and found that 50 μm caffeine caused a significant decrease of PTH secretion and *PTH* gene expression. This decrease occurred in parallel with a decrease of the intracellular cAMP level, protein kinase A activity, and *ADORA1* gene expression, indicating a possible causal relationship. The intracellular level of Ca^2+^ was unaffected even by high concentrations of caffeine. Protein expressions demonstrated two main targets for caffeine—ADORA_1_ and ADORA_2A_.

**Conclusion:**

A physiological high dose of caffeine inhibits PTH secretion in human parathyroid cells, possibly due to a decrease of the intracellular level of cAMP. The observation demonstrates a functional link between caffeine and parathyroid cell function.

Caffeine is the most commonly consumed substance with stimulatory effects on the central nervous system. The main sources are dietary beverages such as coffee, tea, and caffeinated soft drinks. The daily caffeine intake varies largely between countries and individuals, with an average daily consumption above or well above 150 mg in most Western countries (1). Multiple epidemiological and experimental studies have investigated side effects of caffeine and associations to disease phenotypes, such as low bone mineral density (BMD). In vitro studies of osteoblast cultures and in vivo animal studies have shown negative effects of caffeine on osteoblast function, bone matrix formation, and BMD (2–4). Although the results from epidemiological studies are not fully conclusive, many reports found an association between high coffee consumption of more than 3 cups per day and low BMD in women and men, especially when calcium intake was low (5–10).

PTH, vitamin D, and calcitonin are main regulators of calcium homeostasis and bone remodeling involving mainly the kidneys, gut, parathyroid glands, and skeleton. The effects of caffeine on human body functions are not yet fully conclusive. In the kidneys, caffeine intake led to reduced renal reabsorption of calcium (Ca^2+^) without significant change of glomerular filtration (11), which was not fully compensated during nighttime conservation (12), hence giving a net loss of urinary calcium. This calcium loss can be simply overcome by the addition of 1–2 tablespoons milk per cup of coffee (13). In the gut, caffeine intake leads to inhibition of Ca^2+^ absorption (14). PTH, which is secreted from the parathyroid glands, has a direct and positive effect on osteoblast survival and differentiation (15). PTH has been successfully used in the treatment of osteoporosis, and the positive effects on bone formation have been mainly attributed to the increased population of osteoblasts (15). In a Swedish study, a correlation was seen between high coffee consumption and low serum levels of intact PTH in men (16), suggesting a relationship between caffeine and parathyroid gland function, although similar results were not reported in another study of young women (17). However, possible associations between caffeine and PTH secretion from the parathyroid cell have so far not been experimentally explored.

Binding to adenosine receptors (ADORAs) is 1 important mechanism for caffeine action at the cellular level. Four different ADORA types encoded by separate genes are presently known. ADORA_1_ and especially ADORA_2A_ are already significantly activated at a low coffee consumption of 1 cup and are increasingly activated at higher caffeine doses (1). By contrast, ADORA_2B_ and ADORA_3_ are only weakly responsive to caffeine (18). Receptors ADORA_1_ and ADORA_2A_ are G protein-coupled with activating or inactivating effects on adenylyl cyclase, which in turn affects cAMP, protein kinase A (PKA), subsequently regulates cellular functions (18).

Given the observational associations between caffeine, BMD, and PTH, we aimed to explore possible effects of caffeine on PTH secretion from the parathyroid cell. For this purpose, we have applied an established system for studies of human parathyroid cell function in short-term cultures of cells (19–22) from patients with hyperparathyroidism. The effects of caffeine on PTH secretion and *PTH* gene expression, intracellular Ca^2+^ ([Ca^2+^]_i_), cAMP, and PKA activity were assessed as well as expression of ADORA_1_ and ADORA_2A_.

## Patients and Methods

### Patients, parathyroid tissue samples, and cell preparation

Human parathyroid tissue samples were collected with ethical approval at the Karolinska University Hospital, Stockholm, Sweden. Informed consent was obtained from all patients, as documented in the patients' files. Diagnoses were according to the World Health Organization classification (23). Fresh pathological parathyroid cells used in studies of PTH secretion; *PTH*, *ADORA1*, and *ADORA2A* gene expression; intracellular cAMP; and PKA activity assays were from 25 patients (19 females and 6 males) with a median age of 57 years (range, 42–87 y), median serum PTH levels of 361 ng/L (range, 69–1163 ng/L), and median serum ionized calcium levels of 1.46 mm (range, 1.38–1.59 mm). The histopathological diagnoses were primary adenoma (n = 22), lithium-induced hyperplasia or adenoma (n = 2), and secondary parathyroid hyperplasia (n = 1). For physiological studies, fresh parathyroid tissues were randomly collected directly after tissue dissection at surgery, transported in MEM, and isolated into cell clusters by collagenase digestion using previously described methodology (19). To obtain single cells, subsequent digestion in 1× Accutase solution (catalog no. AT104; Innovative Cell Technologies, San Diego, California) was carried out for 6 minutes. Single cells were treated with caffeine within 72 hours after isolation, and samples were collected for analysis of PTH secretion; *PTH*, *ADORA1*, and *ADORA2A* expression; intracellular cAMP level; and PKA activity. In those experiments, omission of caffeine was used as control in each experiment, and its value was set as 1, to which values from caffeine-treated samples were then normalized. Fresh-frozen samples and slides of paraffin-embedded tissues from reference nontumorous parathyroid and parathyroid adenomas (20) were used for Western blot analyses and immunohistochemistry, respectively.

### Measurement of PTH secretion by batch incubation

Cells were isolated and plated (2–5 × 10^5^) in 24-well plates and cultured overnight to allow cells to recover from collagenase digestion and attach to the plate bottom. Before each experiment, cells were preincubated with extracellular solution (EC) containing 1.5 mm Ca^2+^ and 1 mg/mL BSA for 1 hour at 37°C. The medium was then changed to EC containing 1.25 mm Ca^2+^ and 0, 1, 10, or 50 μm caffeine. After 30-minute incubation, medium was collected and directly put on ice. Medium was then centrifuged at 3000 rpm for 5 minutes at 4°C to precipitate cells possibly present in the medium. After removal of the medium, 100 μL Mammalian Protein Extraction Reagent (Thermo Scientific, Hudson, New Hampshire) was added to each well to lyse cells. Protein concentrations were measured using Bio-Rad protein assay. Supernatants were stored at −20°C and quantified for intact PTH using electrochemiluminescence immunoassay (catalog no. 11972103; Roche Diagnostics, Indianapolis, Indiana) at the routine clinical chemistry laboratory in the Karolinska University Hospital, Stockholm, Sweden. For each sample, the PTH value was normalized to the corresponding total protein. Each treatment was performed in duplicate or triplicate. The effects of 50 and 10 μm caffeine were investigated in parallel using cells from the same adenoma. EC with 0.5 and 1.8 mm Ca^2+^ was applied as positive controls for cell responsiveness. For each concentration, 13–26 independent experiments were performed on 5 to 10 patient samples.

### Measurement of [Ca^2+^]_i_ by Fura-2

Measurements of [Ca^2+^]_i_ were carried out using Fura-2 as an indicator, following procedures previously described in detail (19). Isolated cells were grown on glass coverslips overnight until they attached and loaded with 2.5 μm Fura-2 AM in EC solution with 1.25 mm Ca^2+^. Coverslips were placed in a 37°C perfusion chamber exposed to an inverted fluorescence microscope with a ×40 oil objective and connected to a cooled charged-coupled device camera with an imaging system. Cells were stepwise stimulated with 0.5 mm Ca^2+^ and 1.25 mm Ca^2+^, followed by the addition of caffeine at concentrations of 10, 50, 200, 500 μm or 5 mm for 5–10 minutes. Fluorescence was provided to cells with excitation at 340 and 380 nm, and emission was monitored at 505 nm. Three to 4 independent experiments were performed on at least 2 patient samples.

### Protein expression analysis

Western blot analysis was carried out according to previously described methodology for previously published parathyroid tissue samples (20). Total protein extracts from 7 parathyroid chief cell adenomas and 1 biopsy from a normal parathyroid gland were analyzed together with U251 cells used as positive control. The human glioblastoma cell line U251 was kindly provided by Anna-Maria Marino, Department of Clinical Neurosciences, Karolinska Institutet, Stockholm, Sweden. In short, the analysis involved SDS-PAGE, blotting onto nitrocellulose membranes, incubation overnight at 4°C with primary antibodies, followed by incubation with appropriate secondary antibodies. Three primary antibodies were used: rabbit polyclonal anti-ADORA_1_ (catalog no. ab82477; Abcam Inc., Cambridge, Massachusetts) at 1:1000 dilution; rabbit polyclonal anti-ADORA_2A_ (catalog no. ab101678; Abcam Inc.) at 1:800; and anti-GAPDH (catalog no. sc-32233; Santa Cruz Biotechnology, Santa Cruz, California) at 1:5000 as a protein-loading control. Detection was carried out by enhanced chemiluminescence and by exposure to hyperfilm. Immunohistochemistry was carried out on tissue sections from 4 parathyroid adenomas with a normal rim using previously described methods and the anti-ADORA_1_ antibody at a dilution of 1:1000. Incubation without antibody served as negative control.

### Quantitative real-time PCR (qRT-PCR)

Expression of *ADORA1*, *ADORA2A*, and *PTH* was determined in 7 cell culture samples from 5 parathyroid adenomas treated with 0, 1, 10, or 50 μm caffeine at 1.25 mm Ca^2+^ for 30 minutes. Total RNA was extracted using RNeasy Mini Kit (QIAGEN, Valencia, California), quantified by NanoDrop, and reverse transcribed (600 ng) using the High Capacity RNA-to-cDNA Kit (Life Technologies, Gaithersburg, Maryland). cDNA samples (2 μL) were applied for gene expression quantification using TaqMan Universal Master Mix II (Life Technologies) with gene-specific primers and TaqMan probes for *ADORA1* (Hs00379752_m1), *ADORA2A* (Hs00169123_m1), and *PTH* (Hs00757710_g1). Samples were amplified following a standard protocol in an ABI Real-Time PCR 7900HF Fast System (Life Technologies). The housekeeping gene *RPLP0* (Hs00420895_gH) was used as an endogenous control, and a no-template sample was used as a negative control. All samples were loaded in triplicate and run together in a 384-well plate.

### Measurement of intracellular cAMP

Isolated cells were cultured overnight in 24-well plates with 1–2 × 10^5^ cells per well, followed by incubation with caffeine dissolved in 1.25 mm Ca^2+^ EC at concentrations of 0, 1, 10, and 50 μm and 5 mm at 37°C for 30 minutes. Cells incubated with medium without caffeine were used as control. After caffeine treatment, medium was aspirated, and 100 μL 0.1 m HCl was added to the wells, followed by incubation at room temperature for 20 minutes. Cells were collected with a cell scraper and centrifuged at 1000 × *g* for 10 minutes. Supernatant was then acetylated with KOH and acetic anhydride, and cAMP was detected using the cAMP ELA kit (catalog no. 581001; Cayman Chemical Company, Ann Arbor, Michigan) according to the protocol recommended by the manufacturer. Plates were read at 405 nm in a Thermomax microplate reader (Molecular Devices Corp., Sunnyvale, California), and results were analyzed with the 4-Parameter Logistic model in the Masterplex 2010 software (MiraiBio Group, Hitachi Solutions America, Ltd, San Francisco, California). Protein concentrations were measured as described above, and cAMP levels were then normalized to total protein. Independent experiments were repeated 9 times in 5 adenomas.

### PKA activity assay

PKA activity was evaluated using the antibody-based DetectX PKA activity kit (Arbor Assays, Ann Arbor, Michigan) in primary cultured cells treated with 0, 1, 10, or 50 μm caffeine at 1.25 mm Ca^2+^ for 30 minutes. Cells were lysed with 1% Nonidet P-40 lysis buffer containing 0.1% protease inhibitor cocktail, 1 mm phenylmethylsulfonylfluoride, and 10 mm activated Na_3_VO_4_ and assayed according to the protocol of the manufacturer. Horseradish peroxidase conjugate activity was detected by TMB ELISA Substrates system read at 450 nm. PKA activity of each sample was calculated using the 4-Parameter Logistic model in the Masterplex 2010 software and normalized by total protein. Independent experiments were repeated 10 times in 5 adenomas.

### Statistics

Results are given as median (25th, 75th percentiles). Box-plots are used in figures. Statistical significance was analyzed using nonparametric Wilcoxon signed-rank test between 2 groups. A *P* value of less than .05 was considered significant.

## Results

### Inhibition of PTH secretion by caffeine

**Figure 1.**
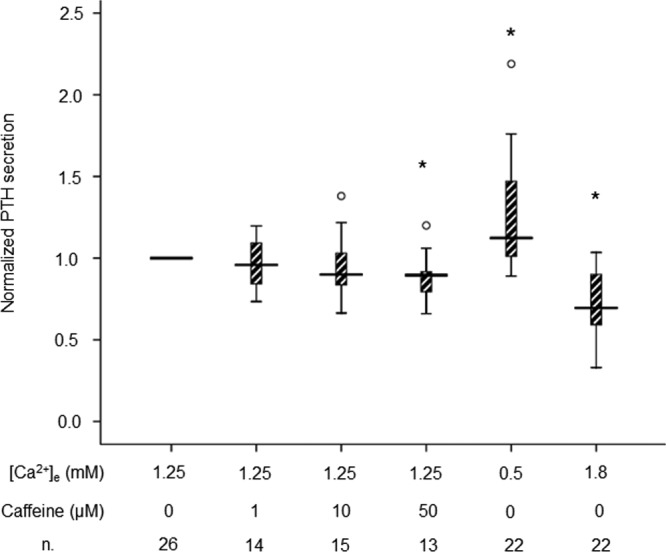
Summary of PTH secretion from human parathyroid cells after caffeine treatment at concentrations of 0, 1, 10, and 50 μm at extracellular calcium ([Ca^2+^]_e_) of 1.25 mm. The PTH level without caffeine was set at 1.0 for each experiment; 0.5 and 1.8 mm Ca^2+^ were applied as controls for cell responsiveness. n, number of experiments. Box-plots show median levels together with 10th, 25th, 75th, and 90th percentiles, outline indicated as open circle. *, *P* < .05 as compared to control cells.

Possible effects of caffeine on PTH secretion were determined by batch incubation studies of human parathyroid adenoma cells. Three physiologically relevant concentrations of caffeine were evaluated: 1, 10, and 50 μm. Caffeine treatment was applied for periods of 30 minutes in each plate. The results are summarized in Figure 1. Treatment with 50 μm caffeine led to a significant inhibition by 10.4% (25th, 75th percentiles: 7.6, 21) of PTH secretion compared with control (*P* < .05). For comparison, 0.5 mm Ca^2+^ caused a 12.4% (0.2, 47.8) increase, whereas 1.8 mm Ca^2+^ led to a 30.5% (9.8, 41.5) decrease of PTH secretion.

### Caffeine does not affect [Ca^2+^]_i_ levels

**Figure 2.**
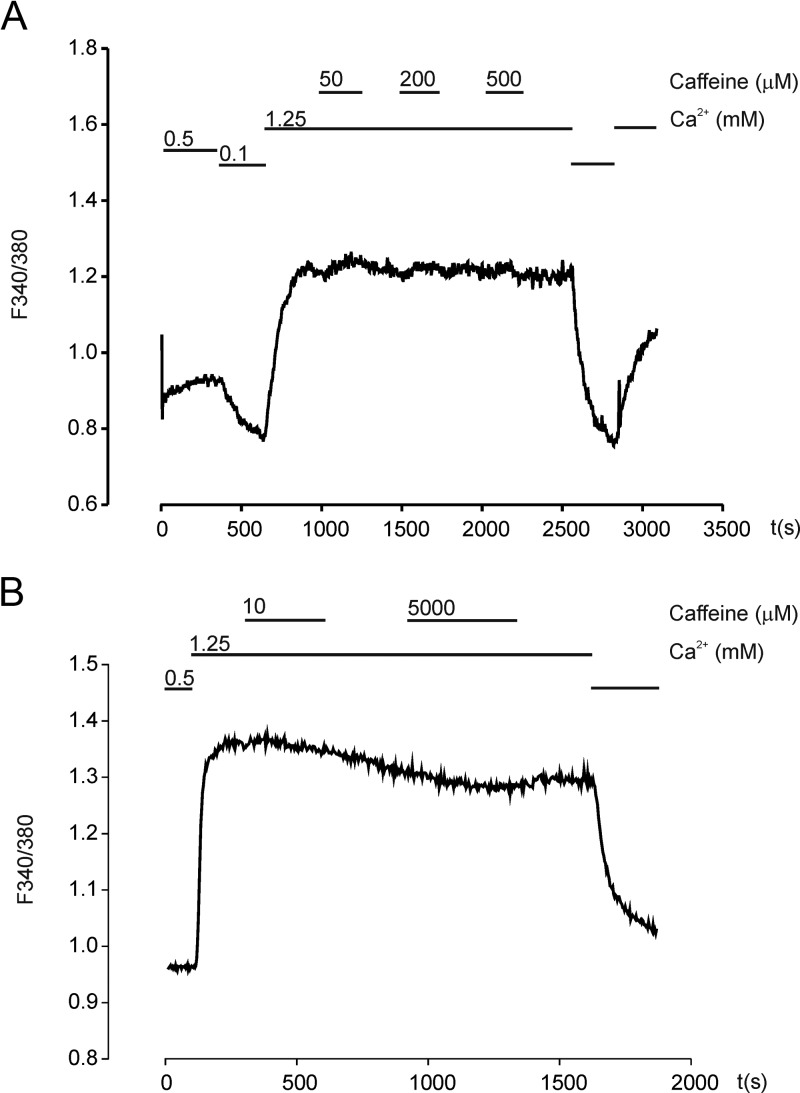
Measurement of [Ca^2+^]_i_ using Fura-2 after application of 50, 200, or 500 μm caffeine (A) and 10 μm and 5 mm caffeine (B).

Possible involvement of altered [Ca^2+^]_i_ levels after caffeine exposure to parathyroid adenoma cells was investigated using the Ca^2+^ indicator Fura-2 and calculation of resulting F_340_/F_380_ ratios as representing the [Ca^2+^]_i_ level. Caffeine was administered in the perfusion system at 1.25 mm Ca^2+^. No significant changes were recorded for F_340_/F_380_ ratios after the addition of caffeine concentrations ranging from 10 μm to 5 mm (Figure 2).

### Expression of ADORA_1_ and ADORA_2A_ in parathyroid tissues

**Figure 3.**
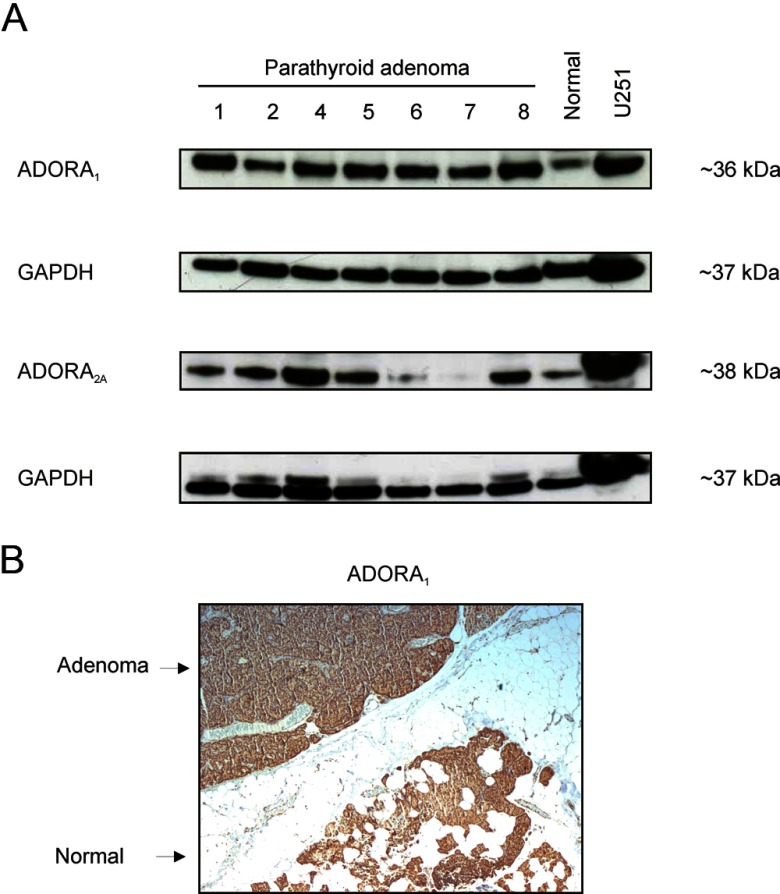
Protein expression of ADORA_1_ and ADORA_2A_ in parathyroid tissues. A, Autoradiograms of Western blot analyses for protein expression of ADORA_1_ and ADORA_2A_ in 7 human parathyroid adenomas, normal parathyroid tissue, and human glioblastoma U251 cells used as positive control. Subsequent incubation with anti-GAPDH was used as protein-loading control. B, Photomicrographs showing ADORA_1_ expression in parathyroid adenoma and in the adjacent rim of normal parathyroid (magnification, ×36).

Expression of the ADORA_1_ and ADORA_2A_ proteins in parathyroid tissues was determined by Western blot analysis. For both antibodies, a product of expected size was detected in the positive control and in parathyroid tissues (Figure 3A). Our results showed that both ADORA_1_ and ADORA_2A_ are present in normal parathyroid tissue. ADORA_1_ showed relatively even expression between the normal parathyroid and the 7 parathyroid adenomas investigated. Protein expression of ADORA_1_ was also demonstrated by immunohistochemistry. As illustrated in Figure 3B, similar expression intensity was observed in adenoma and normal parathyroid rim present on the same slides. However, for ADORA_2A_, varying levels of expression were observed between adenomas (Figure 3A).

### *ADORA1*, *ADORA2A*, and *PTH* gene expressions after caffeine treatment

**Figure 4.**
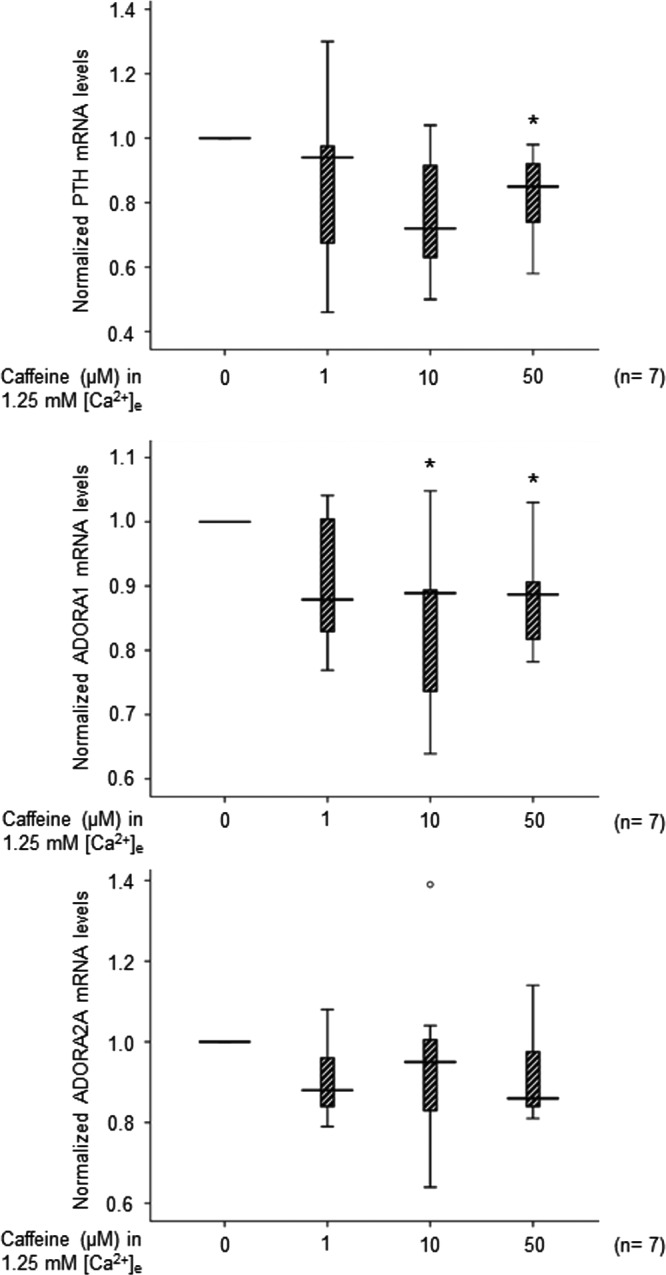
Relative mRNA expression levels of *PTH*, *ADORA1*, and *ADORA2A* determined by qRT-PCR after incubation with 0, 1, 10, or 50 μm caffeine. The expression level in the noncaffeine control was set at the arbitrary value of 1.0 for each experiment. Box-plots show median, 10th, 25th, 75th, and 90th percentiles. *, *P* < .05 as compared to control cells.

The expression of *ADORA1*, *ADORA2A*, and *PTH* after 30-minute caffeine treatment of cultured parathyroid adenoma cells was evaluated by qRT-PCR. A significant decrease of *PTH* mRNA by 15.3% (4.6, 32.4) was found after addition of 50 μm caffeine, but not at 1 or 10 μm (Figure 4). Decrease of *ADORA1* expression was detected at 10 μm caffeine treatment by 11.1% (10.6, 29.7) and after 50 μm by 11% (7.8, 19.4) (Figure 4), but not after 1 μm. However, caffeine did not cause any significant change of *ADORA2A* mRNA level at any tested concentration (Figure 4).

### Effects of caffeine on intracellular cAMP levels and PKA activity

**Figure 5.**
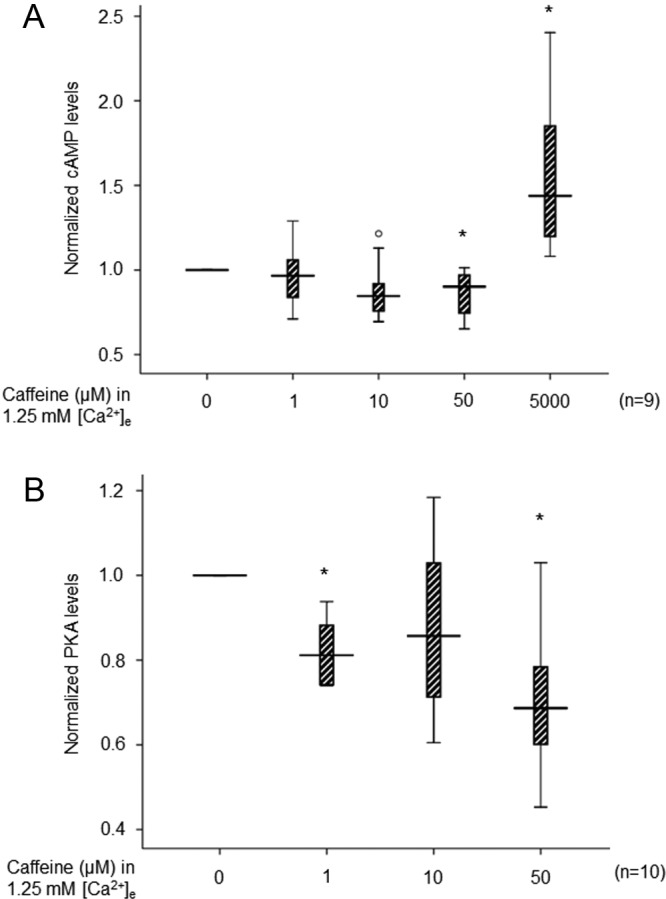
Results from measurements of intracellular cAMP and PKA in human parathyroid adenoma cells exposed to caffeine for 30 minutes. A, Intracellular cAMP measured after administration of caffeine at concentrations of 1, 10, 50 μm and 5 mm, and for control cells without caffeine treatment. B, PKA activities after incubation with 0, 1, 10, or 50 μm caffeine. Intracellular cAMP and PKA activity of the noncaffeine control was set at 1.0 for each experiment. Box-plots show median levels together with 10th, 25th, 75th, and 90th percentiles, outline indicated as open circle. *, *P* < .05 as compared to control cells.

We subsequently determined possible alterations in the levels of cAMP and PKA activity, critical second messengers mediating caffeine cellular function. For this purpose, the content of acetylated cAMP and PKA activity was quantitated after exposure to caffeine in the culturing media for 30 minutes. The results are summarized in Figure 5. The cAMP levels were not significantly affected at 10 μm. A significant decrease of cAMP by 9.8% (2.0, 26.6) was found after caffeine treatment at 50 μm. However, a very high concentration of caffeine (5 mm) was found to significantly elevate the cAMP level by 43.8% (19.8, 90) above control cells (*P* < .05). A significant inhibition of PKA was observed at 1 and 50 μm caffeine by 18.8% (11.6, 18.8) and 31.3% (19.6, 43.3), respectively.

## Discussion

Osteoporosis is a common disease in the elderly population. Bone fracture, which is the main consequence of osteoporosis, leads to decreased quality of life and increased morbidity and mortality. In view of the lack of effective therapies, prevention of osteoporosis is of great interest. Many epidemiology studies have been carried out with the aim to identify risk factors for bone loss (24, 25). Factors like increased age, low body weight, and weight loss have consistently been found to be associated with bone loss. However, possible associations to dietary components, such as caffeine intake, are more obscure. Although some studies did not find an association, other studies reported associations between high coffee intake (more than 3 cups per day that contain 300 mg caffeine) and low BMD (5–9), especially when calcium intake was low.

It is presently unknown how caffeine may influence the bone quality in our body. Some early studies have shown that caffeine intake caused acute increase of urinary calcium excretion (12, 13) and reduced calcium absorption in the gut (14, 15). Cell and animal studies have found that caffeine directly inhibits osteoblast cell function (4) or induces calcium release from bone (26). In the last decade, PTH has been found to be an important factor for bone remodeling, and injection of PTH has been approved for treatment of osteoporosis. Given that both hyper- and hypoparathyroidism are associated with increased risk of bone fracture (27), we speculated that caffeine might affect bone quality through modulation of PTH secretion. Initial data obtained with an alternative technique to study PTH secretion, ie, cell perfusion, revealed a significant inhibition of PTH secretion at nonphysiological high concentrations (200 and 500 μm and 5 mm) of caffeine (data not shown). The present experiments were carried out under physiological conditions; ie, the effect of caffeine was investigated at 1.25 mm Ca^2+^, which is close to the physiological set point for half inhibition of maximal PTH secretion in humans. Our observations included caffeine concentrations of 1, 10, and 50 μm. One to 10 μm corresponds to the serum concentration after the intake of 1 cup (177 mL) of coffee. No significant inhibition of PTH secretion or *PTH* mRNA expression was seen at 1 and 10 μm. Raising the caffeine concentration to 50 μm resulted in significantly decreased PTH secretion by 10.4% and significant inhibition of *PTH* gene expression. Caffeine is quickly absorbed after digestion and easily distributed to the whole body with a half-life of 2.5 to 4.5 hours (1). Therefore, high doses of caffeine intake may inhibit pulsatile secretion of PTH.

In the parathyroid cell, [Ca^2+^]_i_ is a central player for PTH secretion, in which high levels of [Ca^2+^]_i_ result in reduced secretion of PTH. However, even caffeine levels as high as 5 mm did not result in significant changes in [Ca^2+^]_i_ levels. These observations indicate that the inhibitory effect of caffeine on PTH secretion is not due to the elevation of [Ca^2+^]_i_ level. Caffeine at high concentrations is a well-known activator of the ryanodine receptor (RYR), leading to calcium release from sacro/endoplasmic reticulum in many cell types (28, 29). However, it has also been found to reduce inositol trisphosphate (IP_3_)-stimulated calcium release and the following store operated calcium entry (30), which exists in human parathyroid cells (20). The effect of caffeine on [Ca^2+^]_i_ (intracellular calcium) levels varies between cells depending on the distribution of RYR receptors and IP_3_ receptors (26–28). Expression of RYR receptors and IP_3_ receptors in human parathyroid has not been addressed. The lack of altered [Ca^2+^]_i_ after caffeine stimulation may be due to the balance between the two receptors.

cAMP is another important second messenger that mediates caffeine function. Caffeine is known to modulate the intracellular cAMP level through inactivation of the G protein-coupled receptors ADORA_1_ and ADORA_2A_ and inhibition of phosphodiesterase. In parathyroid cells, cAMP has been found to have a positive association with PTH secretion. Several compounds, such as the β-adrenergic agonists isoproterenol and epinephrine and forskolin (31, 32), have been shown to enhance cAMP accumulation and PTH release. In our study, we observed a decrease of cAMP at 50 μm caffeine in parallel with a decrease of PTH secretion. The expected increase of cAMP at a very high concentration of caffeine is most probably a result from inhibition of phosphodiesterase (1). Simultaneously, we observed decreased *ADORA1* gene expression at 10 and 50 μm, whereas no obvious alterations of *ADORA2A* expression were recorded. We detected both ADORA_1_ and ADORA_2A_ at the protein level in normal parathyroid and parathyroid adenomas. ADORA_1_ showed an equal expression in normal and parathyroid adenomas, whereas ADORA_2A_ was found to be weak or absent in some parathyroid adenoma samples. Increased or unaltered *ADORA1* mRNA expression has been reported in brain after long-term caffeine treatment (33, 34). Other studies also found that short-term caffeine treatment reduces adenylyl cyclase activity, whereas chronic caffeine treatment results in increased adenylyl cyclase activity due to adaptation after caffeine intake (35). The decrease of *ADORA1* expression observed in this study may be due to the short-term caffeine incubation. Once cAMP is produced, it activates PKA and mediates multiple cellular functions. In our study, we found that PKA activity was decreased in parallel with a decrease of cAMP after administration of 50 μm caffeine.

In conclusion, our results show that a high concentration of caffeine causes inhibition of PTH secretion in human parathyroid cells. This effect may be due to inhibition of intracellular cAMP and the PKA pathway, but it does not involve alterations of [Ca^2+^]_i_. Our study is the first to investigate the effect of caffeine on PTH secretion in human parathyroid cells. The observation demonstrates a functional link between caffeine and parathyroid cell function.
